# Cell-level metadata are indispensable for documenting single-cell sequencing datasets

**DOI:** 10.1371/journal.pbio.3001077

**Published:** 2021-05-04

**Authors:** Sidhant Puntambekar, Jay R. Hesselberth, Kent A. Riemondy, Rui Fu

**Affiliations:** 1 RNA Bioscience Initiative, University of Colorado School of Medicine, Aurora, Colorado, United States of America; 2 University of Colorado, Boulder, Colorado, United States of America; 3 Department of Biochemistry and Molecular Genetics, University of Colorado School of Medicine, Aurora, Colorado, United States of America; IMBA, AUSTRIA

## Abstract

Single-cell RNA sequencing (scRNA-seq) provides an unprecedented view of cellular diversity of biological systems. However, across the thousands of publications and datasets generated using this technology, we estimate that only a minority (<25%) of studies provide cell-level metadata information containing identified cell types and related findings of the published dataset. Metadata omission hinders reproduction, exploration, validation, and knowledge transfer and is a common problem across journals, data repositories, and publication dates. We encourage investigators, reviewers, journals, and data repositories to improve their standards and ensure proper documentation of these valuable datasets.

## Abstract 2

Most Gene Expression Omnibus (GEO) depositions of single-cell mRNA sequencing data do not include cell-level metadata generated by typical analysis pipelines; this Essay maintains that this omission greatly hinders reproduction, exploration, validation, and knowledge transfer and is a common problem across journals, data repositories, and publication dates.

Single-cell RNA sequencing (scRNA-seq) has empowered discoveries of cell heterogeneity and state transitions at unprecedented resolution and throughput. New technological developments have broadened the scope of measurable molecules, extending beyond RNA to measure cell surface proteins [1,2]. Every single experiment potentially generates thousands to millions of cell transcriptomes spanning diverse cell types, subtypes, transition phases, or perturbed states, so increasing effort has been applied to the reanalysis of published datasets. The large amount of public data provide a rich resource for comparative analysis between and within cell types, and for building databases of consensus cell types based on molecular profiles [3–5].

Single-cell data analysis has become increasingly user-friendly. However, much of the analysis time is devoted to tuning unsupervised clustering parameters and assigning clusters to a particular cell type. This is a crucial step in the analysis to determine whether cell type annotations are congruent with previously characterized cell types and to justify the discovery of novel ones. Many tools have been developed to simplify cell type annotation by comparing new single-cell datasets to existing reference single-cell datasets [6], so that known cell types can be assigned to clusters in an automated fashion based on the similarity of their gene-expression profiles to cell types in public single-cell datasets [7–10]. Additionally, scRNA-seq batch-correction methods, such as Seurat’s integration method, fastMNN, and Harmony, enable fine-grained reanalysis and comparison of published scRNA-seq datasets at the individual cell level [11–13]. Another popular reanalysis method uses marker genes or gene signatures for each cell type to generate gene-set module scores for each cell [14,15]. However, reanalysis of single-cell datasets using these methods requires proper documentation of the cell types present in the reference dataset to provide interpretable comparisons between the query datasets and reference publication data.

## Reporting the minimal data necessary to replicate cell types identified in single-cell datasets

**Fig 1:**
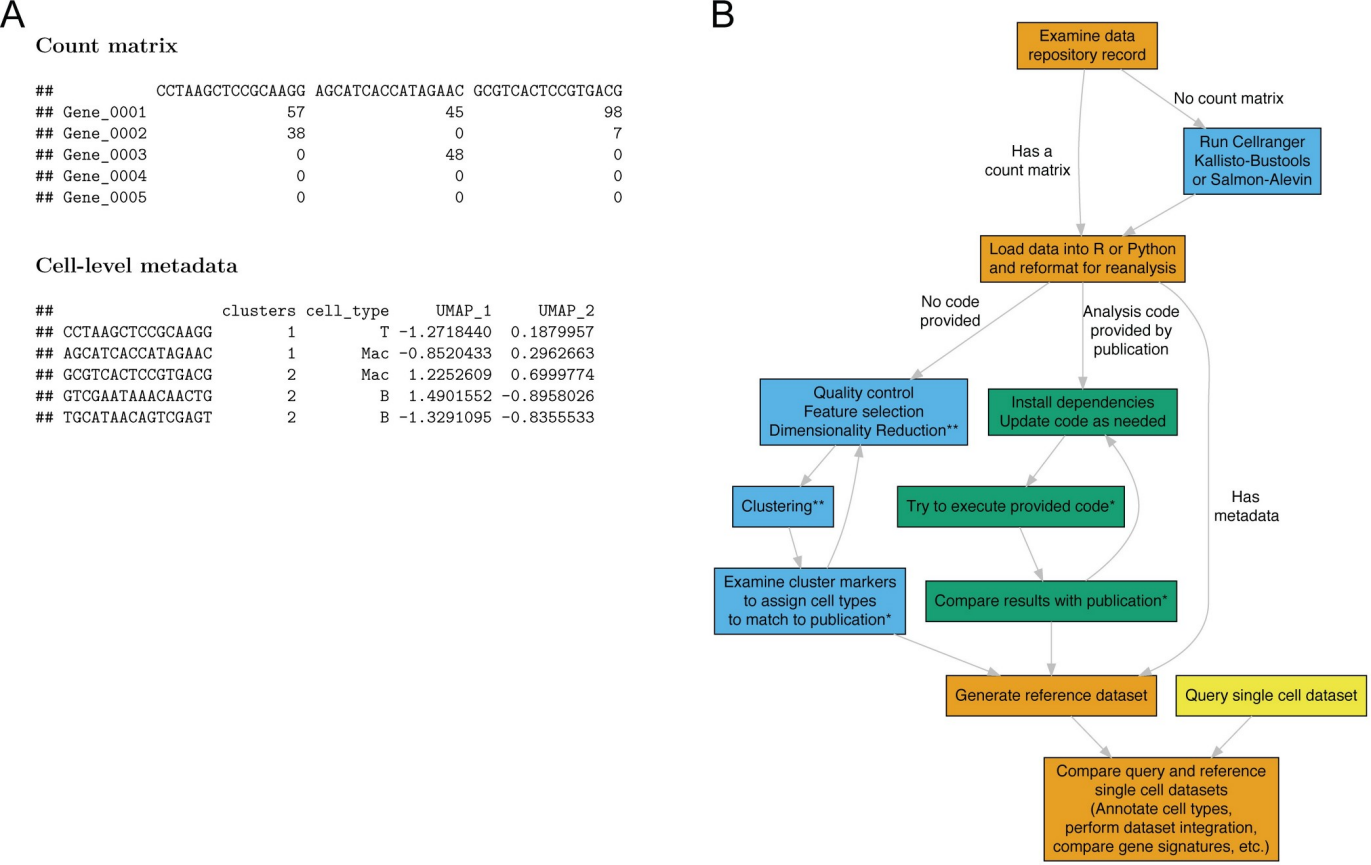
Processed data files necessary for replicating single-cell studies. (A) Example of a gene-by-cell count matrix containing single-cell measurements and a cell-level metadata table containing annotations inferred from the analysis of the single-cell dataset. (B) Workflow of analysis steps for regenerating cell type or gene-expression signatures from public datasets for comparative analysis of single-cell datasets. * indicates a step requiring an analyst to make subjective decisions; ** indicates a step that often includes a nondeterministic algorithm.

Replicating the transcriptomes of cell types described in a published scRNA-seq dataset at minimum requires 2 pieces of data: a count matrix and a table of cell-level metadata (**Fig 1A**). The count matrix quantifies RNA abundance in each cell, which most typically takes the form of a matrix with genes as rows, cell identifiers as columns, and integer counts of observed RNA molecules. The cell identifiers are generally DNA barcodes indicating single droplets (10x Genomics or DropSeq) or sample identifiers indicating a well or chamber with a single cell captured in well-based methods (Smart-Seq2). The count matrix is commonly generated by software pipelines such as Cellranger from 10X Genomics, Kallisto-Bustools, or Alevin [16–18].

The second piece of data, a metadata table, contains cell-level annotations describing the inferred cell type and other descriptive information for each cell identifier present in the count matrix. Cell-level metadata are defined based on information learned from the analysis and depends on the specific analyses conducted by the analyst, which makes these annotations unlikely to be replicated by an automated software pipeline. It also contrasts with sample-level metadata (e.g., sequence library type, cell isolation method, sequencing instrument), which describe the experimental procedures used to generate the data. Data repositories, such as GEO and ArrayExpress, were built at a time when one sequencing library or microarray chip generally only contained data for one biological sample. Each sample is the subject of analysis and is described by sample-level metadata to identify the biological sample. This annotation framework can provide adequate documentation for single-cell studies using low-throughput well-based methods such as Smart-Seq2, where each cell is considered a single sample and processed as an independent sequencing library. However, single-cell studies using popular droplet-based platforms such as the 10x Genomics chromium or Drop-Seq now generate thousands of cells per biological sample and sequencing library. Cell-level annotations therefore do not conform to the sample-level documentation framework, and the depositor must provide this information as an additional processed file.

Cell-level annotations are generated during analysis by software suites, such as Seurat, scater, or Scanpy, and can include cluster assignments from unsupervised clustering algorithms, cell type assignments inferred from automated methods or manual inspection of gene expression signatures by experts in the field, and additional cell-level attributes such as inferred cell cycle stage [10,19,20]. The cluster or cell-type assignments are a critical piece of information as these assignments identify the exact cells that are compared in differential gene expression tests, allowing users to replicate marker genes or gene expression signatures described in a study. The coordinates of dimensionality reductions (PCA, UMAP, tSNE) can also be included as cell-level metadata. These coordinates allow users to replicate dimension reduction projections, which are frequently the most common visualization in scRNA-seq publications but are also not guaranteed to be reproduced upon reanalysis.

In the absence of per-cell metadata, the effort, time, and field-specific expertise required to compare cell subpopulations described in a publication to new single-cell datasets is dramatically increased. Instead of easily leveraging peer-reviewed expertise contained in the cell-level metadata, researchers are forced to rerun pipelines, which can take several hours, and scour the original text for a handful of marker genes described to assign cell type/states subjectively.

Even with careful reanalysis, several factors can limit the original study’s reproducibility if data have to pass through the entire analysis pipeline (**Fig 1B**). First, the exact parameters used for the data processing and analysis are rarely fully reported in a manuscript. Subtle differences in algorithm or parameter choices can lead to different downstream results. For example, during quality control, the algorithm selected for distinguishing cell-containing from empty droplets influences which cell populations are retained in the downstream analysis and can lead to excluding cells with low RNA content [21]. Second, multiple steps in the analysis rely on nondeterministic algorithms, including the results from clustering and dimensionality reduction. The output of these algorithms cannot be guaranteed to be reproducible across operating systems and software versions. Finally, rapid development in the scRNA-seq software field leads to inevitable deprecation of outdated functionalities and possibly silent yet impactful alterations to the underlying algorithms. Due to these potential pitfalls, even inclusion of original analysis code, while also valuable and should be encouraged, is a poor replacement for generated cell-level metadata.

## Public single-cell RNA-seq datasets frequently omit cell-level metadata necessary for reanalysis

In an effort to curate reference atlases of diverse cell types, we attempted to identify cell-by-gene count matrices and associated cell-level metadata from single-cell studies in public data repositories. We found that many studies failed to provide cell-level annotations for the deposited data. To determine how frequently studies contain cell-level annotations, we queried the Gene Expression Omnibus (GEO), which is the most commonly used data repository for single-cell studies (used by 78.1% of studies with public data in a curated database of single-cell studies) [22]. To assess the extent of missing cell-level annotations, we crafted a custom query string to recover single-cell experiments because there is no specific annotation that can be used to identify single-cell datasets in GEO. A query string of "*expression profiling by high throughput sequencing" AND ("single nuclei" OR "single cell" OR "scRNAseq" OR "scRNA-seq" OR "snRNAseq" OR "snRNA-seq")* coupled with further keyword filtering using the GEOquery R package returned 3,902 GEO entries (after merging GEO SuperSeries). These included 97.4% of the GEO studies previously manually curated in Svensson and colleagues, supporting the performance of our query [22]. We then programmatically identified supplemental files with names containing common terminology associated with cell-level metadata reporting, “meta,” “annot,” “type,” “clustering,” and “colData,” as well as R and python-readable data formats "rds,” “rda,” “rdata,” “loom,” and “h5ad.” Only 13.5% of GEO entries contain cell-level metadata (19.7% for entries within the Svensson and colleagues–curated database).

To confirm the accuracy of our classification approach, we performed manual inspection of 173 randomly selected studies that we identified as single-cell datasets through querying GEO. We found that 9.8% of studies that we classified as single-cell datasets were instead other sequencing modalities (e.g., bulk RNA-seq), highlighting the importance of having standardized metadata terms to identify single-cell sequencing datasets. Of the remaining true single-cell studies, 6.4% (10/156) contained metadata files that were missed by our automated classification, while 88.9% (24/27) of called-positive cases truly contained cell-level metadata (and 22/27 contained actual cell type information). Based on this analysis, we estimate that at most 25% of studies deposited in GEO contain cell-level annotations. This number is comparable to analyses we conducted on ArrayExpress records, where we estimate that 15% of scRNA-seq datasets generated from the 10x Genomics platform deposited cell-level annotations. GEO records do not have single cell–specific library preparation metadata terms (e.g., Smart-Seq2, Drop-Seq, Fluidigm-C1), which limited our ability to programmatically identify studies that deposited each cell as an independent record. These studies may have included relevant cell-level annotations; however, the absence of a standardized metadata term (e.g., cell-type) prevented systematic examination of the annotations in these records.

**Fig 2:**
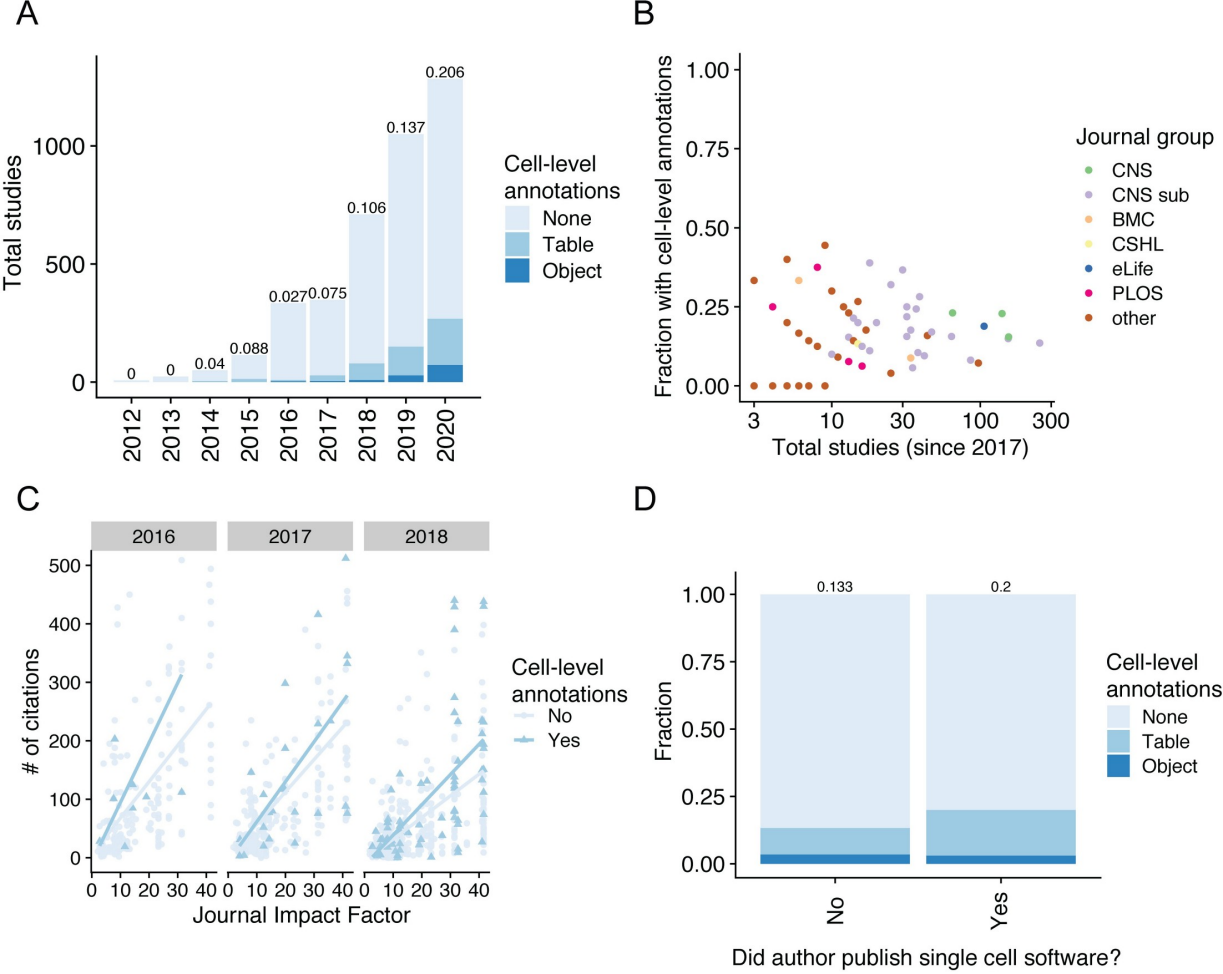
The majority of single-cell sequencing datasets archived on GEO do not have cell-level annotations. (A) Number of single-cell datasets in GEO annotated with the proportion that contain cell-level metadata per year, either as plain text tables or binary objects. (B) Fraction of studies published in each group of journals compared to the total number of studies published by each group. (C) Comparison of the number of citations for studies containing or lacking cell-level metadata in 2016, 2017, or 2018. (D) Fraction of studies, since 2017, containing cell-level annotations published by authors with a previous publication of a single cell–related software tool. The numerical data underlying plots may be found at https://github.com/rnabioco/someta/tree/master/inst/manuscript and http://doi.org/10.5281/zenodo.4695069.

Further exploring the GEO entries with publication information linked through GEO and PubMed, we found that the percentage of metadata-containing entries have slowly improved with time, as pipeline standards matured and awareness of this issue has grown. However, even for studies published in 2020, the fraction with metadata remains at 20.6% (**Fig 2A**). In addition, the issue is widespread through journals of every family and tier (**Fig 2B**). While enforcement of data deposition through journals has been highly effective at improving data accessibility, once again the lack of specific guidelines towards scRNA-seq supporting information hurts the overall goal.

Next, to corroborate with our own data analysis experience, we explored whether publications with annotated per-cell metadata potentially lead to more citations by facilitating minimal-effort comparison of reported data and cell type gene-expression signatures to new experiments (**Fig 2C**). Without rigorous statistical testing, due to the limited number of metadata-containing studies and the numerous confounding factors affecting citations, we note that we observe a general trend encouraging the habit of presenting cell metadata. We also examined datasets deposited by authors on publications describing scRNA-seq informatics tools. Tools developed by these authors generally require cell-level metadata, and therefore we hypothesized that associated publications would be more likely to include cell-level metadata. We identified these authors by querying software curated by the scRNA-tools database [23] and discovered that GEO entries with contribution from these authors tend to have better, yet still limited, cell metadata deposition (**Fig 2D**).

## Suggestions for improving documentation of scRNA-seq datasets

We believe that the lack of cell-level annotations severely limits the reanalysis of public datasets and that addressing this problem will require a community-wide effort, from authors to journals and data repositories (**Box 1**). Primary analysts, who are responsible for conducting the single-cell analysis, are aware of the importance of cell-level annotations for single-cell analysis, as they are necessary to generate many of the figures presented in manuscripts. When preparing a dataset for deposition, they should consider whether the processed data deposited are sufficient for replicating the gene expression profiles of the cell types described in their studies. Common scRNA-seq analysis data structures all incorporate cell-level annotations in a table-like format, either as an R data.frame or a pandas DataFrame in Python (**Fig 1A**) which are easily exported as comma or tab-separated text files along with the count matrix. An example of a well-documented dataset, GEO accession GSE137710, contains a metadata file for each sample (e.g., GSE137710_human_melanoma_cell_metadata_9315x14.tsv.gz) with cell-level annotations identifying the cell type (e.g., “b_cell,” “melanoma,” “myeloid,” “T/NK”) [24]. Each cell barcode is annotated with the cell type described in the study, which enables very rapid (<5 minutes) downstream analyses to compare expression patterns and markers for these newly described cell types to other single-cell datasets. Another example, ArrayExpress record E-MTAB-6701, which characterized cells at the fetal maternal interface, includes 2 processed files with descriptive names (raw_data_10x.txt and meta_10x.txt), identifying the count matrix and cell-level metadata, respectively [25]. Because there is no standard file format or naming convention for these data, using common language to describe these files will greatly aid in their discovery via manual searches or programmatic API calls. Lastly, analysts should request cell-level metadata from lead authors when it is not available in public repositories and encourage them to update their records with these data.

A growing number of researchers are actively promoting reproducibility and data exploration by presenting interactive data browsers or hosting code and metadata files on open-access repositories such as GitHub. These interactive resources can enable researchers without extensive programming experience to explore single-cell datasets. However, not all popular cell browser solutions offer metadata export, and external datasets not linked and documented in standardized repositories such GEO or ArrayExpress are difficult to navigate. Additionally, public data browsers may require periodic maintenance to ensure their availability and therefore are at risk of becoming inaccessible.

Peer review can also help to improve documentation of single-cell datasets. Reviewers of single-cell studies should request access to the GEO or ArrayExpress records and examine the deposited processed data files. Cell-level metadata tables are generally small simple text files that can be rapidly examined to see if they contain cell-level annotations describing the cell types in the study. Journals can also assist in improving documentation standards by encouraging reviewers to examine deposited data and by providing language specific to single-cell studies to recommend inclusion of cell-level metadata in datasets described in a publication. In cases where annotation changes occurred through the revision process, journals could consider sending reminders to update deposition records with the latest cell-level metadata at the time of manuscript acceptance.

**Box 1. Recommendations for depositing scRNA-seq datasets**

For investigators and reviewers

1. Require that analysts provide a metadata table containing cell-level metadata and a count matrix with RNA abundance measurements. The cell-level metadata should contain the cell identifiers present in the matrix and provide the inferred cell-type or other cell-level annotations described in the associated publication. A binary object saved from the analysis framework could also be supplied (e.g.,.rds for R or.h5ad for Python).
2. When reviewing single-cell sequencing studies, ensure that the authors have deposited the proper cell-level metadata alongside the raw data into a suitable repository (e.g., GEO, ArrayExpress).
3. Encourage previous depositors of single-cell sequencing data to update their records with cell-level metadata, if it was not included in the original submission.

For journals

1. Include language about requirements/recommendations for external single-cell datasets to contain proper cell-level metadata.
2. Ask reviewers to review material deposited to external data repositories.

For data repositories

1. Introduce a standardized annotation specifying that the dataset contains single-cell data. For GEO, commonly used single-cell sequencing methods could be added to the library strategy annotation (e.g., scRNA-seq, snRNA-seq, CITE-seq, etc.).
2. Updating submission guidelines and examples to require metadata with cell-level annotations for single-cell dataset submissions. For GEO, this would be accomplished by updating the “Processed data files” requirements to outline required data types for single-cell sequencing submissions (**Fig 1A**).

“*For single-cell sequencing data*, *in addition to standard count matrices (genes-by-cells)*, *we expect users to deposit metadata with cell-level annotations generated during the course of analysis*.*”*

Data repositories could improve standards by providing guidelines for appropriate documentation and highlighting example datasets. Currently, the only scRNA-seq-specific requirement noted in the current GEO guideline is for raw data deposition (https://www.ncbi.nlm.nih.gov/geo/info/seq.html). The requirements for supplemental processed data files are vague and do not reference commonly generated single-cell data files. ArrayExpress recently adopted new standards for single-cell dataset deposition, which provide documentation on how to annotate the “inferred cell type” identified in the experiment. However, these guidelines are only defined for well-based methods, where each cell is considered a single sample. For droplet-based methods, there is no clear guidance on the content or file format to include to annotate the cell types inferred from the experiment. We believe that the absence of single cell–specific guidelines, example datasets, or recommendations of file formats for processed data has led to many studies only depositing the cell-by-gene count matrix to satisfy the minimum processed data requirements for data submissions.

## Moving forward

Single-cell sequencing datasets have rapidly grown in number and complexity, with thousands of datasets ranging up to millions of cells, providing a wealth of new information about cell types and cell states. We hope that improved standards for public data deposition will encourage large-scale archiving and integration efforts for single-cell datasets akin to the efforts of databases such as Recount2 generated for bulk sequencing methods [26]. Efforts to produce single-cell atlases from public datasets will continue to require time-consuming curation in a study-by-study manner until the deposition of machine-readable standardized annotation files becomes common practice in the community. In this pursuit, cell-level annotation reporting is merely the first necessary step of many, including enforced naming conventions of files, file formats, platform names, and controlled vocabulary for cell types [27], all of which will improve data accessibility, reproducibility, and reuse of published results.

We do not intend to provide the single-cell sequencing equivalent of microarray standards (e.g., MIAME, MINSEQE) [28], which has been recently explored in detail [29] but aim to highlight this troubling issue, to encourage adoption of reproducible data deposition practices [30], and to promote discussion of best practices within the community (**Box 1**). Large-scale efforts to curate cell atlases are currently underway in the Human Cell Atlas, Allen Brain institute, and Fly Cell Atlas, and we hope that the standards implemented in these consortia can contribute to the development of best practices for documenting single-cell datasets in the wider community. A guided and standardized effort will facilitate scientific transparency and communication and require minimal additional work on the part of authors.

## Materials and methods

Analysis code is available on GitHub (https://github.com/rnabioco/someta). The repository automatically monitors missing cell metadata and periodically generates updated reports. With each completed automated analysis, the latest version of combined data are available on GitHub as an RDS object and at https://raysinensis.shinyapps.io/clustifyr-web-app/?tab=someta for interactive explorations. The numerical data underlying plots and quantification mentioned in text, concerning scRNA-seq dataset identification overlap with Svensson and colleagues, manual spot check results, and arrayexpress metadata analysis, may be found at https://github.com/rnabioco/someta/tree/master/inst/manuscript and http://doi.org/10.5281/zenodo.4695069.

### GEO query and parsing

GEO snapshot of December 31, 2020 was obtained via NCBI E-utility calls using a query string of *"expression profiling by high throughput sequencing" AND ("single nuclei" OR "single cell" OR "scRNAseq" OR "scRNA-seq" OR "snRNAseq" OR "snRNA-seq")*. Series returned by this query were further analyzed with the GEOquery R package, including further filtering of all descriptive fields by keywords listed above, merging subseries from superseries into a single series where applicable, and extraction of supplemental files names [31].

### Programmatic identification of cell metadata files

To determine which GEO entries contain cell annotation metadata, the following assumptions were made: (1) a stand-alone metadata file should contain “meta,” “annot,” “type,” “clustering,” or “coldata” (case-insensitive) in its file name; (2) metadata can also be housed in R and python-readable data formats with the extensions of "rds,” “rda,” “rdata,” “loom,” or “h5ad.” These target strings were determined from common terminology from analysis suites and experience in navigating scRNA-seq records and corroborated by multiple researchers involved in examining GEO records. In manual inspections, the most common inaccuracies with these assumptions are: annotation files containing gene annotations rather than cell-level metadata, data objects containing other data rather than Seurat/SingleCellExperiment/Scanpy objects, and well-based samples using sample names or sample metadata fields to indicate cell type annotation. These inaccuracies are difficult to identify with automated code and highlight the need for better standardization in the field.

### Additional publication and journal-level analyses

For GEO entries providing linked PubMed IDs, additional publication information was retrieved using R packages easyPubMed and rcrossref [32,33]. Cases where the journal name from PubMed is incompatible with rcrossref records were manually fixed before downstream analysis in R. For analysis of scRNA-seq bioinformatic tool authors, scRNA-tools database and R package rbiorxiv were used [23,34].

### ArrayExpress query

Initial query of datasets was conducted through ArrayExpress R package, using the recommended term “RNA-seq of coding RNA from single cells.” We extracted the “library construction” annotation field and focused on datasets generated with the 10x Genomics platforms, as these are cases where sample metadata cannot provide per-cell annotations. Associated file names were extracted with custom code from IDF and SDRF files of each entry, and then subjected to the programmatic identification of cell metadata files process used for GEO query as above.

## References

[1] Stoeckius M, Hafemeister C, Stephenson W, Houck-Loomis B, Chattopadhyay PK, Swerdlow H, et al. Simultaneous epitope and transcriptome measurement in single cells. Nat Methods. 2017;14:865–8. 10.1038/nmeth.4380 28759029 PMC5669064

[2] Setliff I, Shiakolas AR, Pilewski KA, Murji AA, Mapengo RE, Janowska K, et al. High-throughput mapping of B cell receptor sequences to antigen specificity. Cell. 2019;179:1636–1646.e15. 10.1016/j.cell.2019.11.003 31787378 PMC7158953

[3] Cao Z-J, Wei L, Lu S, Yang D-C, Gao G. Searching large-scale scRNA-seq databases via unbiased cell embedding with Cell BLAST. Nat Commun. 2020;11:3458. 10.1038/s41467-020-17281-7 32651388 PMC7351785

[4] Franzén O, Gan L-M, Björkegren JLM. PanglaoDB: a web server for exploration of mouse and human single-cell RNA sequencing data. Database. 2019. 10.1093/database/baz046 30951143 PMC6450036

[5] Mori T, Shinwari N, Fujibuchi W. scMontage: Fast and Robust Gene Expression Similarity Search for Massive Single-cell Data. 2020. p. 2020.08.30.271395. 10.1101/2020.08.30.271395

[6] Abdelaal T, Michielsen L, Cats D, Hoogduin D, Mei H, Reinders MJT, et al. A comparison of automatic cell identification methods for single-cell RNA sequencing data. Genome Biol. 2019;20:194. 10.1186/s13059-019-1795-z 31500660 PMC6734286

[7] Kiselev VY, Yiu A. Hemberg M. scmap: projection of single-cell RNA-seq data across data sets. Nat Methods. 2018;15:359–62. 10.1038/nmeth.4644 29608555

[8] Aran D, Looney AP, Liu L, Wu E, Fong V, Hsu A, et al. Reference-based analysis of lung single-cell sequencing reveals a transitional profibrotic macrophage. Nat Immunol. 2019;20:163–72. 10.1038/s41590-018-0276-y 30643263 PMC6340744

[9] Fu R, Gillen AE, Sheridan RM, Tian C, Daya M, Hao Y, et al. clustifyr: An R package for automated single-cell RNA sequencing cluster classification. F1000research. 2020;9:223. 10.12688/f1000research.22969.2 32765839 PMC7383722

[10] Butler A, Hoffman P, Smibert P, Papalexi E, Satija R. Integrating single-cell transcriptomic data across different conditions, technologies, and species. Nat Biotechnol. 2018;36:411–20. 10.1038/nbt.4096 29608179 PMC6700744

[11] Haghverdi L, Lun ATL, Morgan MD, Marioni JC. Batch effects in single-cell RNA-sequencing data are corrected by matching mutual nearest neighbors. Nat Biotechnol. 2018;36:421–7. 10.1038/nbt.4091 29608177 PMC6152897

[12] Tran HTN, Ang KS, Chevrier M, Zhang X, Lee NYS, Goh M, et al. A benchmark of batch-effect correction methods for single-cell RNA sequencing data. Genome Biol. 2020;21:12. 10.1186/s13059-019-1850-9 31948481 PMC6964114

[13] Korsunsky I, Millard N, Fan J, Slowikowski K, Zhang F, Wei K, et al. Fast, sensitive and accurate integration of single-cell data with Harmony. Nat Methods. 2019;16:1289–96. 10.1038/s41592-019-0619-0 31740819 PMC6884693

[14] Aibar S, González-Blas CB, Moerman T, Huynh-Thu VA, Imrichova H, Hulselmans G, et al. SCENIC: single-cell regulatory network inference and clustering. Nat Methods. 2017;14:1083–6. 10.1038/nmeth.4463 28991892 PMC5937676

[15] Tirosh I, Izar B, Prakadan SM, Wadsworth MH 2nd, Treacy D, Trombetta JJ, et al. Dissecting the multicellular ecosystem of metastatic melanoma by single-cell RNA-seq. Science 2016;352:189–196. 10.1126/science.aad0501 27124452 PMC4944528

[16] Melsted P, Sina Booeshaghi A, Gao F, Beltrame E, Lu L, Hjorleifsson KE, et al. Modular and efficient pre-processing of single-cell RNA-seq. 2019. p. 673285. 10.1101/673285

[17] Srivastava A, Malik L, Smith T, Sudbery I, Patro R. Alevin efficiently estimates accurate gene abundances from dscRNA-seq data. Genome Biol. 2019;20:65. 10.1186/s13059-019-1670-y 30917859 PMC6437997

[18] Zheng GXY, Terry JM, Belgrader P, Ryvkin P, Bent ZW, Wilson R, et al. Massively parallel digital transcriptional profiling of single cells. Nat Commun. 2017;8:ncomms14049. 10.1038/ncomms14049 28091601 PMC5241818

[19] McCarthy DJ, Campbell KR, Lun ATL, Wills QF. Scater: pre-processing, quality control, normalization and visualization of single-cell RNA-seq data in R. Bioinformatics. 2017; btw777. 10.1093/bioinformatics/btw777 28088763 PMC5408845

[20] Wolf FA, Angerer P, Theis FJSCANPY. large-scale single-cell gene expression data analysis. Genome Biol. 2018;19. 10.1186/s13059-017-1382-0 29409532 PMC5802054

[21] Lun ATL, Riesenfeld S, Andrews T, Dao TP, Gomes T. participants in the 1st Human Cell Atlas Jamboree, et al. EmptyDrops: distinguishing cells from empty droplets in droplet-based single-cell RNA sequencing data. Genome Biol. 2019;20:63. 10.1186/s13059-019-1662-y 30902100 PMC6431044

[22] Svensson V, da Veiga Beltrame E. Pachter L. A curated database reveals trends in single-cell transcriptomics. Database (Oxford). 2020; 2020. 10.1093/database/baaa073 33247933 PMC7698659

[23] Zappia L, Phipson B, Oshlack A. Exploring the single-cell RNA-seq analysis landscape with the scRNA-tools database. PLoS Comput Biol. 2018;14:e1006245. 10.1371/journal.pcbi.1006245 29939984 PMC6034903

[24] Brown CC, Gudjonson H, Pritykin Y, Deep D, Lavallée V-P, Mendoza A, et al. Transcriptional Basis of Mouse and Human Dendritic Cell Heterogeneity. Cell. 2019;179:846–863.e24. 10.1016/j.cell.2019.09.035 31668803 PMC6838684

[25] Vento-Tormo R, Efremova M, Botting RA, Turco MY, Vento-Tormo M, Meyer KB, et al. Single-cell reconstruction of the early maternal-fetal interface in humans. Nature. 2018;563:347–53. 10.1038/s41586-018-0698-6 30429548 PMC7612850

[26] Collado-Torres L, Nellore A, Kammers K, Ellis SE, Taub MA, Hansen KD, et al. Reproducible RNA-seq analysis using recount2. Nat Biotechnol. 2017;35:319–21. 10.1038/nbt.3838 28398307 PMC6742427

[27] Jupp S, Burdett T, Malone J, Leroy C, Pearce M, Mc Murry J, et al. A New Ontology Lookup Service at EMBL-EBI. Proceedings of SWAT4LS International Conference. CEUR-WS.org; 2015. Available from: http://ceur-ws.org/Vol-1546/paper_29.pdf.

[28] Edgar R, Barrett T. NCBI GEO standards and services for microarray data. Nat Biotechnol. 2006:1471–2. 10.1038/nbt1206-1471 17160034 PMC2270403

[29] Füllgrabe A, George N, Green M, Nejad P, Aronow B, Fexova SK, et al. Guidelines for reporting single-cell RNA-seq experiments. Nat Biotechnol. 2020. 10.1038/s41587-020-00744-z 33188371 PMC9302581

[30] Larsson O, Sandberg R. Lack of correct data format and comparability limits future integrative microarray research. Nat Biotechnol. 2006;24:1322–3. 10.1038/nbt1106-1322 17093466

[31] Davis S, Meltzer PS. GEOquery: a bridge between the Gene Expression Omnibus (GEO) and BioConductor. Bioinformatics. 2007:1846–7. 10.1093/bioinformatics/btm254 17496320

[32] Chamberlain S, Boettiger C, Hart T, Ram K. rcrossref: Client for various ‘CrossRef APIs.’. 2016.

[33] Fantini D. easyPubMed: Search and Retrieve Scientific Publication Records from PubMed. 2019.

[34] Fraser N. R client for interacting with the “bioRxiv” API. 13 Jul 2020 [cited 2020 Nov 19]. Available from: https://CRAN.R-project.org/package=rbiorxiv.

